# Decidual CD8^+^T cells exhibit both residency and tolerance signatures modulated by decidual stromal cells

**DOI:** 10.1186/s12967-020-02371-3

**Published:** 2020-06-01

**Authors:** Lu Liu, Xixi Huang, Chunfang Xu, Chunqin Chen, Weijie Zhao, Dajin Li, Liping Li, Li Wang, Meirong Du

**Affiliations:** 1 Laboratory for Reproductive Immunology, NHC Key Lab of Reproduction Regulation (Shanghai Institute of Planned Parenthood Research), Shanghai Key Laboratory of Female Reproductive Endocrine Related Diseases, Hospital of Obstetrics and Gynecology, Fudan University Shanghai Medical College, Shanghai, People’s Republic of China; 2 Department of Obstetrics and Gynecology, Guangzhou First People’s Hospital, School of Medicine, South China University of Technology, Guangzhou, People’s Republic of China

**Keywords:** Maternal–fetal tolerance, CD8^+^T cell, Tissue resident memory cell, T cell exhaustion

## Abstract

**Background:**

During early pregnancy, tolerance of the semi-allogeneic fetus necessitates comprehensive modifications of the maternal immune system. How decidual CD8^+^T (CD8^+^dT) cells balance maternal tolerance of the fetus with defense from invading pathogens remains undefined.

**Methods:**

We investigated the distribution patterns of CD8^+^T cells and their heterogeneity in paired peripheral blood and decidual tissue in the first trimester of pregnancy using flow cytometry and mRNA-Seq. Gene Set Enrichment Analysis was utilized to determine the transcriptional features of CD8^+^dT cells. Moreover, we examined activation of T cells when they were cocultured with trophoblasts, in addition to the effect of the fetal–maternal environment on peripheral CD8^+^T (CD8^+^pT) cells.

**Results:**

We found that, compared with CD8^+^pT cells, CD8^+^dT cells consisted mainly of effector memory cells (T_EM_) and terminally differentiated effector memory cells (T_EMRA_). Both T_EM_ and T_EMRA_ subsets contained increased numbers of CD27^+^CD28^−^ cells, which have been shown to possess only partial effector functions. In-depth analysis of the gene-expression profiles of CD8^+^dT cells revealed significant enrichment in T cell exhaustion-related genes and core tissue residency signature genes that have been found recently to be shared by tissue resident memory cells and tumor^−^infiltrating lymphocytes (TILs). In accordance with gene expression, protein levels of the exhaustion-related molecules PD-1 and CD39 and the tissue resident molecules CD103 and CXCR3 were increased significantly with almost no perforin secretion in CD8^+^dT cells compared with CD8^+^pT cells. However, the levels of granzyme B, IFN-γ, and IL-4 in CD8^+^dT cells were increased significantly compared with those in CD8^+^pT cells. Both CD8^+^dT and CD8^+^pT cells were not activated after being cocultured with autologous trophoblast cells. Moreover, the production of granzyme B in CD103^+^CD8^+^dT cells decreased significantly compared with that in their CD103^−^ counterparts. Coculture with decidual stromal cells and trophoblasts upregulated CD103 expression significantly in CD8^+^pT cells.

**Conclusions:**

Our findings indicate that the selective silencing of effector functions of resident CD8^+^dT cells may favor maternal–fetal tolerance and that the decidual microenvironment plays an important role in promoting the residency of CD8^+^T cells and their tolerance–defense balance.

## Background

Maternal–fetal immune tolerance is a classic immuno-paradox that has puzzled researchers for almost 70 years [1]. CD8^+^T cells are one of the main components of adaptive immunity and are key mediators of allograft rejection [2, 3]. CD8^+^T cells that infiltrate decidual tissue are an important component of local immune cells, and they are likely to interact directly with allogenic trophoblast cells that invade the maternal decidua [4–6]. Therefore, elucidating the mechanisms by which decidual CD8^+^T (CD8^+^dT) cells balance the competing needs for fetal tolerance and protection from invading pathogens is critical in understanding either early pregnancy failure or mid–late pregnancy complications, and it may also impact the studies on allograft transplantation and cancer.

As has been well-established, the maternal immune system can recognize fetal antigens, even though fetal trophoblast cells, which are in direct contact with the maternal immune system, exhibit aberrant HLA expression [4, 6, 7]. However, the question of whether CD8^+^dT cells can either elicit effector responses or become totally dysfunctional and exhibit impaired effector functions is still unresolved. The differentiation status of CD8^+^T cells and their corresponding functions have been studied extensively, and many phenotypic markers have been identified to categorize these cells [8, 9], and used to describe CD8^+^dT cells of term pregnancy [10]. However, the heterogeneity of CD8^+^dT cells during early pregnancy has not been completely elucidated. T cell exhaustion is studied widely in conditions associated with tumor and chronic virus infection, wherein either dysfunctional or exhausted CD8^+^T cells are activated initially and gain effector functions and then ultimately become dysfunctional after chronic exposure to antigens in a proinflammatory environment [11, 12]. Decidual T cells may likely share the same signatures because of consistent fetal antigen exposure. T cell dysfunction was initially detected in chronic lymphocytic choriomeningitis virus infection in mice, where virus cannot be eliminated by CD8^+^T cells [13]. In humans, T cell dysfunction has been demonstrated in a wide variety of cancers that go out of control and persist in the human body. A series of coinhibitory molecules (e.g., PD-1, CD160, and LAG-3) that can impair T cell function, have been studied to identify dysfunctional T cells [14, 15]. The differentiation status of CD8^+^dT cells and whether they are either fully dysfunctional or retain the ability to elicit effector responses remain unclear.

The tissue resident memory T cell (T_RM_) is a new type of effector memory cell that has been identified recently in studies focused on tissue infiltrating lymphocytes, and expression of CD69 and CD103 are attributed to their residency. T_RM_ constitutes a first line of defense against pathogens that invade epithelial and mucosal tissues. Phenotypic analysis of CD8^+^dT cells showed a population of CD8^+^CD103^+^T cells in decidual tissue [16], and an altered endometrial CD69^+^CD103^+^CD8^+^ T cell population was found in recurrent miscarriage [17], their studies provide clues for the tissue residency of uterine infiltrating CD8^+^ T cells. As of yet, the residency signatures of decidual infiltrating CD8^+^T cells have not been studied. Though the tumor microenvironment has been demonstrated to be hostile to T cell functions [18–20], the question of whether the interactions between the fetal–maternal interface microenvironment and CD8^+^pT cells can promote their residency remains unknown. The objectives of our study were to investigate the detailed phenotypic and transcriptional characteristics of decidua-derived CD8 + T cells to comprehensively understand their functional features at maternal–fetal interface. Our findings show that decidua is enriched with activated effector memory T cells that were selectively dysfunctional and demonstrated tissue residency signatures. Moreover, CD8^+^dT cells cannot be activated by autologous trophoblast cells, whereas decidual stromal cells (DSCs) can promote T cell tissue residency.

## Methods

### Human samples

Discarded human placental and decidual materials from the first trimester of pregnancy were obtained from healthy women (gestational age 6–12 weeks, n = 64) undergoing elective termination of pregnancy at the Obstetrics and Gynecology Hospital, Fudan University. In addition, the peripheral blood leukocytes were collected at the same time. Written informed consent was obtained from all participants. The overall study was reviewed and approved by the Human Research Ethics Committee of the Obstetrics and Gynecology Hospital, Fudan University (Approval number: Kyy2016-4).

### Isolation of mononuclear cells from the decidua and peripheral blood

Decidual lymphocytes and peripheral blood leukocytes were isolated as previously described [21, 22]. Decidual tissues of the first trimester were identified and separated from villous tissues. The collected decidual tissues were washed, minced, and then digested with 1.0 mg/ml collagenase type IV (Worthington Biomedical, U.S.A.), and 300 μg/ml DNase I (Sigma–Aldrich, UK) for 40–60 min at 37 °C. After digestion, cells were washed and filtered through 100-, 70-, and 40-μm sieves. Cells were re-suspended in phosphate-buffered saline (PBS), layered on a discontinuous Percoll density gradient (20%/40%/60%; GE Healthcare, U.S.A.) and centrifuged for 20 min at 800×*g*. DSCs were isolated from the 20%/40% Percoll interface, whereas lymphocytes were isolated from the 40%/60% Percoll interface, and both were washed twice in PBS. Peripheral blood mononuclear cells (PBMC) were isolated using Ficoll (GE Healthcare, U.S.A.) density gradient centrifugation (20 min, 800×*g*).

### Sorting of CD8^+^ T cells

The isolated mononuclear lymphocytes from both the decidua and peripheral blood were directly stained for sorting. For FACS sorting, they were incubated with conjugated mouse anti^−^human antibodies, including anti-CD3 FITC (BioLegend, UK), anti-CD56 PerCp/Cy5.5 (BioLegend, UK), anti-CD4 BV421 (BD Biosciences, U.S.A.), and anti-CD8a PE/Cy7 (BioLegend, UK), for 30 min at 4 °C. After incubation, they were treated with LIVE/DEAD^®^ Fixable Aqua Dead Cell Stain (Invitrogen Life Technologies, U.S.A.) for 10 min. CD3^**+**^CD56^−^CD4^−^CD8^**+**^ cells were sorted on a BD FACS Aria-II machine to obtain a purity > 95%. For coculturing with either DSCs or trophoblast cells, CD8^+^T cells from the decidua and PBMC via magnetic affinity cell were sorted using CD8 MicroBeads (MiltenyiBiotec, Germany), according to the manufacturer’s instructions.

### RNA preparation and mRNA-Seq

The total RNA of sorted CD8^+^T cells from the decidua and peripheral blood was lysed and extracted using the RNeasy Mini Kit (Qiagen, Germany) according to the manufacturers’ instructions. Purity and integrity of the extracted RNA was checked on an Agilent Bioanalyzer 2100 (Agilent Technologies, U.S.A.). The library preparation, clustering, and sequencing have been described in a previous study [23]. The prepared libraries were sequenced on an Illumina Hi-seq 2500 platform.

### mRNA-Seq data analysis

Sequenced reads were aligned to the human reference genome using the STAR software package [24]. Exons from all isoforms of a gene were merged to create one meta-gene. The number of reads falling in the exons of this meta-gene was counted using HTSeq-count, and differential expression analysis was conducted using DE-Seq [25]. Differences in gene expression with a p value of < 0.05 (paired *t* test) were considered significant. Volcano Plot and Heatmap analysis of differential genes was performed by using the online gene set enrichment analysis (GSEA) [26].

### Flow cytometry

Cell surface and intracellular molecular expressions were evaluated by flow cytometry using CytoFLEX (BeckmanCoulter, U.S.A.). Fluorescein-conjugated mouse anti-human antibodies were used, including CD3-Alexa Fluor 700, CD3-BV650, CD8-BV786, CD8-PerCP/Cy5.5, CD45RA-APC/CY7, CCR7-PE/CY7,CD27-PE, CD28-BV421, CD69-APC/CY7, CD103-BV605, CXCR3-BV510, HLA-DR-APC, CD39-BV421, PD-1-PE, CD127-PE/CY7, CD62L-PE/CY7, Perforin-APC, Granzyme B-PE, IFN-γ-PE, and IL-4-APC (Biolegend, UK). For cell-surface staining, single-cell suspensions were stained on ice for 30 min in PBS with 1% fetal bovine serum (FBS). For intracellular staining, cells were fixed and permeabilized using the Fix/Perm kit (BD Biosciences, U.S.A.). To detect intracellular cytokines, CD8^+^T cells were stimulated for 6 h with phorbol 12-myristate 13-acetate (PMA; 1 μg/mL; Sigma) and ionomycin (1 μg/mL; Sigma), and 4 h with GolgiStop (1 μL/mL; BD Biosciences) in a round-bottom 96-well plate. Thereafter, cells were harvested, stained for surface expression, and then fixed and permeabilized for intracellular staining. Flow cytometry data was analyzed using FlowJo software (BD, UK) and CytoExpert software (Beckman Coulter, U.S.A.).

### Isolation of trophoblast cells

Trophoblast cells were isolated as previously described [27, 28]. First trimester villous tissue was gently scraped from the basal membrane, and immersed in a solution of trypsin (0.2%) and 0.1 mg/ml DNase I for 8 min at 37 °C. The trypsin was quenched with an F12 medium containing 20% FBS and 1% Pen/Strep (HyClone, U.S.A.) and filtered through 100-, 70-, and 40-μm sieves. The digestion procedure was repeated three times. Cells were washed and layered on a discontinuous Percoll density gradient (35%/60%; GE Healthcare), and centrifuged at 800×*g* for 20 min. Cells were collected, washed, and incubated in a 30-mm tissue culture dish at 37 °C for 20 min to remove macrophages. The purity of isolated trophoblasts was tested via flow cytometry as previously described [29]. Trophoblasts were then seeded in 96-well culture plate (50,000 per well; Costar) precoated with Matrigel (Corning, U.S.A.).

### Cell coculture experiments

Trophoblasts were cultured in a DMEM/F12 medium (HyClone, U.S.A.) containing 20% FBS. CD8^+^T cells (5 × 10^4^ cells/well) were added to coculture with the trophoblasts for 24 or 72 h. In selected experiments, isolated DSCs were seeded in a 24-well culture plate (10^5^ cells/well; Costar), and cultured in DMEM/F12 media (HyClone, U.S.A.) containing 10% FBS. Peripheral CD8^+^ T cells (10^5^ cells/well) were added 24 h later, and cocultured with DSCs for 72 h.

### Statistical analysis

Flow cytometry data was analyzed using FlowJo software (BD, UK) and CytoExpert software (Beckman Coulter, U.S.A.). All statistical analyses and data plotting were performed using the Graphpad Prism software (v8.0, GraphPad Software Inc., U.S.A.). Statistical analysis between groups was conducted using the paired *t*-*test*, and *p* value of< 0.05 was considered significant.

## Results

### Different gene-expression profile between decidual CD8^+^T cells and peripheral CD8^+^T cells

**Fig. 1:**
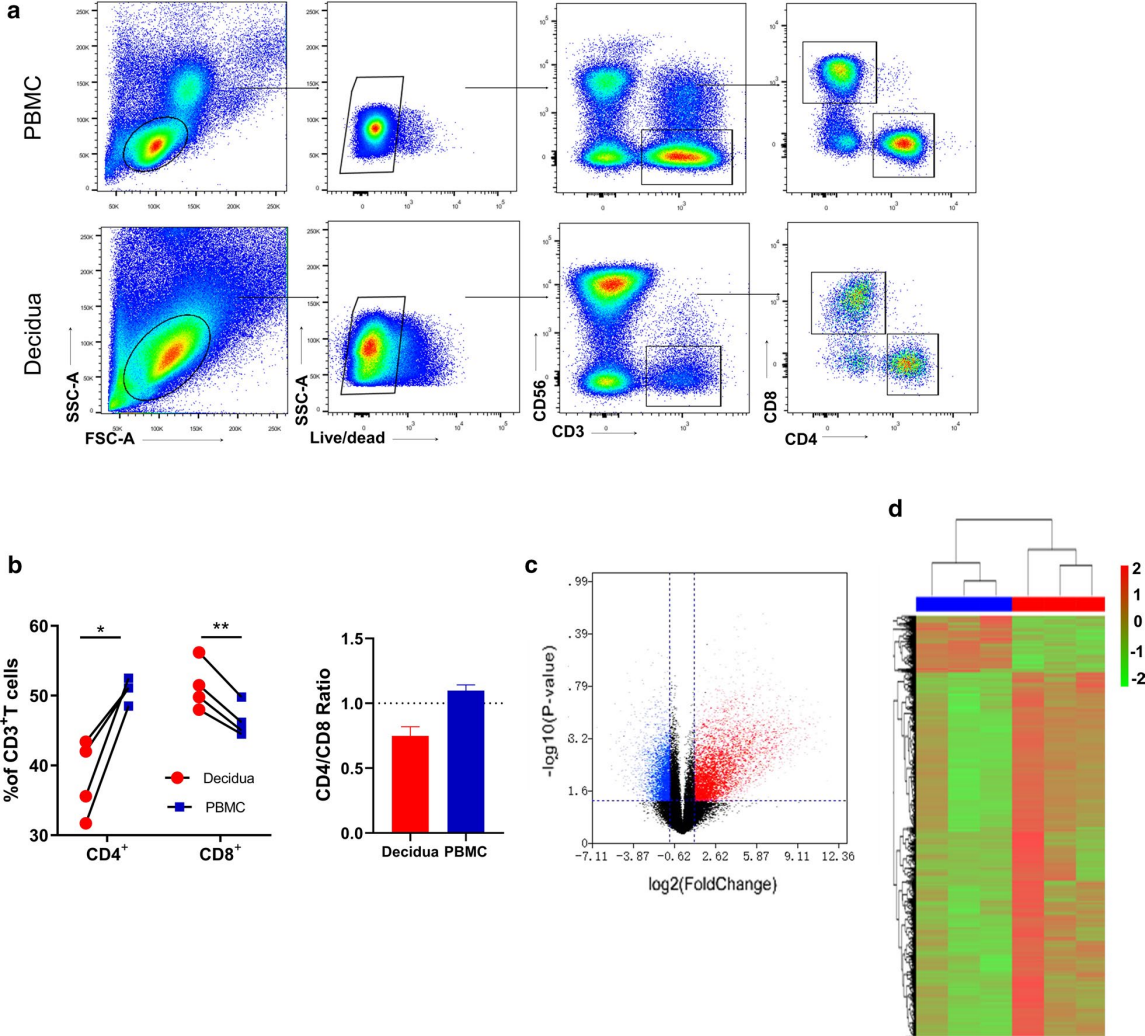
Transcriptional analysis of CD8^+^ T cells from peripheral blood and decidua. **a** Gating strategy of CD8^+^T cells from maternal peripheral blood and decidua, CD8^+^T cells were gated from live CD3^+^, CD56^−^, and CD4^−^ cells. Fluorescence-activated cell sorting was performed to purify the CD8^+^T cells by this gating strategy; **b** Left: Percentage of CD4^+^T cells and CD8^+^T cells in CD3^+^T cells from paired PBMC and decidua. n = 4, paired *t*^−^*test*, *p = <0.05 **p = <0.01; Right: Ratio of frequencies of CD4 versus CD8 from PBMC and decidua. **c** Volcano plot representation of differential expression analysis of genes; Red and blue points mark the genes with significantly increased or decreased expression, respectively, in samples of CD8^+^dT cells compared with samples of CD8^+^pT cells (FDR < 0.01). The x-axis shows fold-changes (log2) in expression, and the y-axis shows the log *p* value of a gene being expressed differentially. In both data sets, Smchd1 is the top-ranked gene. Blue dots are downregulated genes, and red dots are upregulated genes, of CD8^+^dT cells compared with CD8^+^pT cells; **d** Heatmap result of an unsupervised hierarchical clustering of genes that is significantly different (*p *< 0.01) in CD8^+^dT cell samples compared with CD8^+^pT cell samples. Each column represents a patient (blue: CD8^+^pT, red: CD8^+^dT), and each row represents a gene. The heatmap indicates the level of row normalized gene expression. Red = high expression; Green = low expression

Decidual CD8 + T (CD8^+^dT) cells are the main candidates to recognize and respond to fetal antigens at the maternal–fetal interface. To assess the signatures of gene expression in decidual CD8^+^ T cells of the first trimester, we performed high-throughput mRNA-Seq for paired CD8^+^dT cells and peripheral CD8^+^ T cells (CD8^+^pT) from the same participant. T cells were purified via fluorescence-activated cell sorting; the gating strategy is shown in Fig. 1a. We sorted CD4^−^CD8^**+**^ cells from live CD3^+^CD56^−^ populations. At the same time, frequencies of CD4^+^ and CD8^+^ cells in CD3^+^ T cells were compared between the decidua and peripheral blood (Fig. 1b). In the decidua, the frequency of CD8^+^ T cells in CD3^+^ T cells was approximately 48%–56.2%; this was significantly higher than that in CD4^+^ T cells (36.2%–49.8%). In peripheral blood, the frequency of CD8^+^ T cells (36.2–49.8%) was significantly lower than that of CD4^+^T cells (48.5%–52.5%). CD4/CD8 ratio in decidual tissue or peripheral blood further demonstrated the significance of frequency of CD8^+^ T vs CD4^+^ T cells in decidual tissue (Fig. 1b). We demonstrated the differentially expressed genes between CD8^+^ dT cells and CD8^+^ pT cells by means of a volcano plot and a heatmap (Fig. 1c, d). We observed a total of 8100 differentially expressed genes, with 5212 genes being upregulated and 2888 genes being downregulated in CD8^+^dT cells compared with CD8^+^pT cells. The preferentially accumulated CD8^+^ T cells in the decidua with upregulated gene-expression profile suggest that they constitute an important part of fetal–maternal interface, and are actively engaged in fetal tolerance.

### Decidual CD8^+^ T cells consist mainly of the CD27^+^CD28^+^ effector memory subtype

**Fig. 2:**
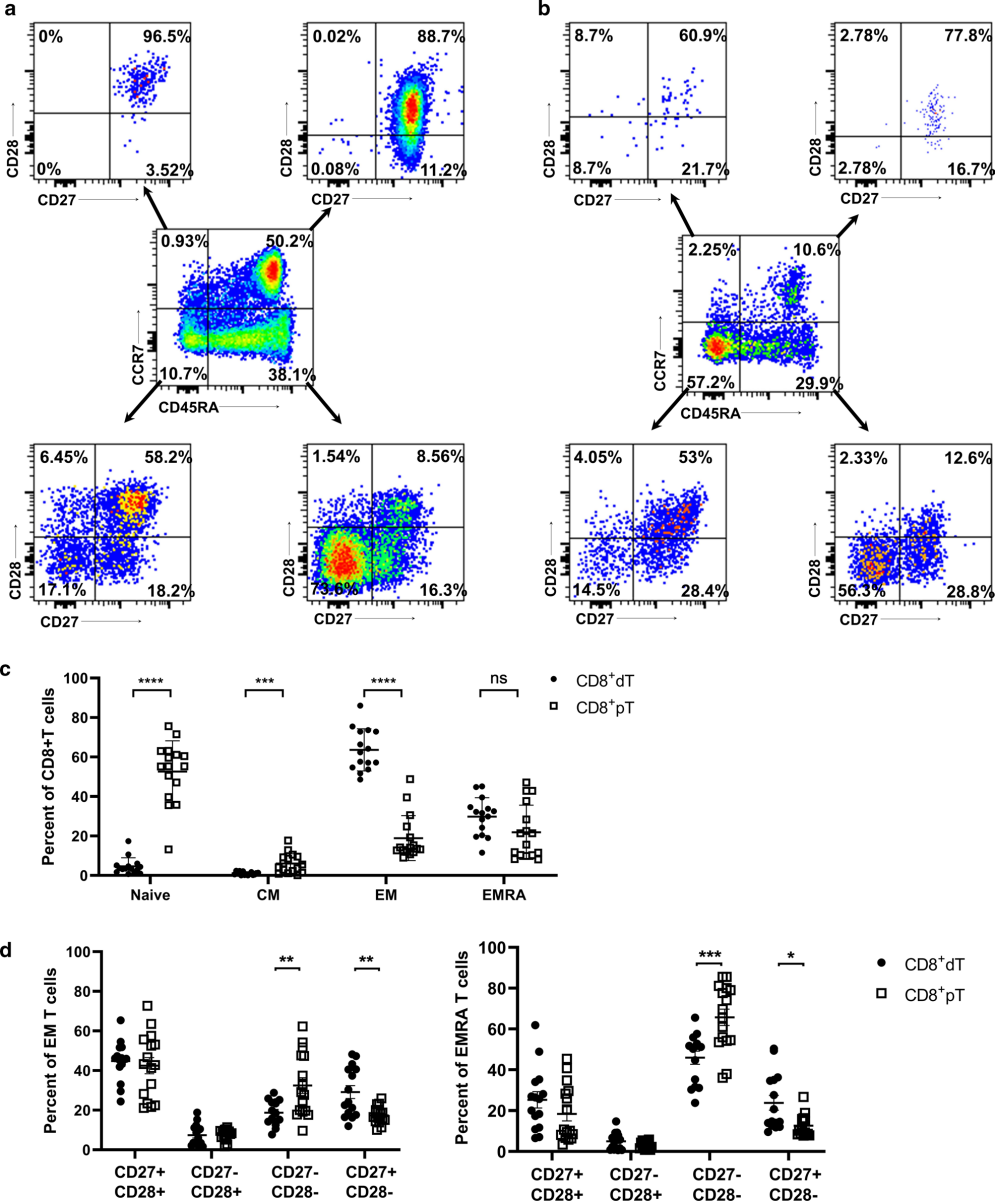
Comparison of differentiation status of CD8^+^dT cells and CD8^+^pT cells. The gating strategy of CD8^+^T cells from maternal peripheral blood (**a**) and decidua (**b**). The memory phenotype was evaluated by expression of CD45RA and CCR7, and each of the CCR7 versus CD45RA dot plot quadrants were further analyzed for CD27 versus CD28 staining. **c** Cell surface markers CD45RA and CCR7 were used to classify naive (CD45RA^+^CCR7^+^), terminally differentiated effector memory (EFF or TEMRA; CD45RA^+^/CCR7^−^), effector-memory (EM; CD45RA^−^CCR7^−^), and central-memory (CM; CD45RA^−^CCR7^+^) CD8^+^ T cells. **d** The proportion of each subset of CD8^+^dT cells and CD8^+^pT cells. n = 15, paired *t*^−^*test*, *p = < 0.05, **p = < 0.01, ***p = <0.005, ****p = <0.0001

CD8^+^ T cell differentiation process has been extensively studied during viral infections [30–32]. These studies have identified several phenotypic markers to categorize these cells and particularly CD8 + T cell subsets capable of eliciting a cytotoxic response. To identify the differentiation status of decidual CD8^+^ T cells during the first trimester, we used the cell surface markers CD45RA and CCR7 to classify naïve (CD45RA^+^CCR7^+^), terminally differentiated effector (T_EMRA_; CD45RA^+^/CCR7^−^), effector memory (T_EM_; CD45RA^−^CCR7^−^), and central memory (T_CM_; CD45RA^−^CCR7^+^) CD8^+^T cells in decidual tissues (Fig. 2a, b). The dominant population of CD8^+^dT cells was the T_EM_ subset, which constituted 48.6%–75.5% of the total decidual CD8^+^T cells; this was markedly higher compared with CD8^+^pT cells, where the proportion was 9.1%–39.5%. The second dominant population of CD8^+^ dT cells was the TEMRA, a subset often related to terminal T cell differentiation, which constituted 11.6%–45.1% of CD8^+^T cells, comparable to the case with CD8^+^pT cells (8.1%–47.1%) (Fig. 2c). Furthermore, to determine additional heterogeneity in CD8^+^T cells [33], we subdivided the main T cell subsets according to the expression of CD27 and CD28 (Fig. 2a, b). CD27 and CD28 are costimulatory receptors that are involved in the generation of Ag-primed cells and regulation of T cell activation, respectively [34, 35]. As shown in Additional file 1: Figure S1a, naïve cells in both CD8^+^dT cells and CD8^+^pT cells consisted exclusively of CD27^+^CD28^+^ cells, demonstrating that they comprised less differentiated subsets. T_CM_ cells displayed similar phenotypes to naïve T cells. However, decidual and peripheral T_EM_ cells comprised more highly differentiated subsets, with lower frequencies of CD27^+^CD28^+^ cells and higher frequencies of CD27^−^CD28^−^ and CD27^+^CD28^−^ cells (Fig. 2d). Furthermore, the percentages of CD27^−^CD28^−^ double negative T_EM_ and T_EMRA_ cells significantly reduced, whereas the percentages of CD27^+^ single positive T_EM_ and T_EMRA_ cells were significantly increased in CD8^+^dT cells compared to CD8^+^pT cells (Fig. 2d). These data suggest that CD8^+^dT cells may pursue alternative means of T_EM_ and T_EMRA_ cell differentiation in relation to CD8^+^pT cells.

### Highly differentiated decidual CD8^+^ T dells enriched core tissue residency and T cell exhaustion transcriptional signatures

We have shown that decidual CD8^+^ T cells showed more upregulated genes and highly differentiated status compared to peripheral CD8^+^ T cells. To further identify molecular and functional features of CD8^+^dT cells in early pregnancy, we used GSEA to compare the genes of CD8^+^dT cells and CD8^+^pT cells with existing KEGG (Kyoto Encyclopedia of Genes and Genomes) gene sets in the ImmSigDB database. We demonstrated that genes related to glycolytic metabolism, the mTOR signaling pathway, and the T cell receptor signaling pathway were upregulated significantly in CD8^+^dT cells compared with those in CD8^+^pT cells (Table 1), suggesting that CD8^+^dT cells undergo metabolic reprogramming during T cell activation. Moreover, GSEA showed that expression of genes associated with the granzyme pathway was increased significantly in CD8^+^ dT cells compared with CD8^+^pT cells (Table 1), indicating increased effector function of CD8^+^dT cells. In addition, CD8^+^dT cells were significantly enriched in genes related to tissue homing (leukocyte transendothelial migration) and the chemokine signaling pathway (Table 1).

**Table 1:** GSEA analysis of upregulated genes in decidua CD8^+^T cells

Gene set	Set size	ES	NES	P value	FDR value
Glycolysis_gluconeogenesis	165	0.431	1.945	< 0.0001	0.014
Purine_metabolism	149	0.54	1.87	< 0.0001	0.05
Regulation_of_autophagy	21	0.634	1.816	< 0.0001	0.05
MTOR_signaling_pathway	50	0.581	1.815	< 0.0001	0.05
TGF_beta_signaling_pathway	84	0.657	1.691	< 0.0001	0.05
Leukocyte_transendothelial_migration	111	0.634	1.686	< 0.0001	0.05
T_cell_receptor_signaling_pathway	106	0.35	1.66	< 0.0001	0.05
Chemokine_signaling_pathway	179	0.55	1.61	< 0.0001	0.06
Granzyme pathway	1173	0.393	1.52	< 0.0001	0.022

*ES* enrichment score, *NES* normalized enrichment score, *FDR* false discovery rate, adjusted p value

**Fig. 3:**
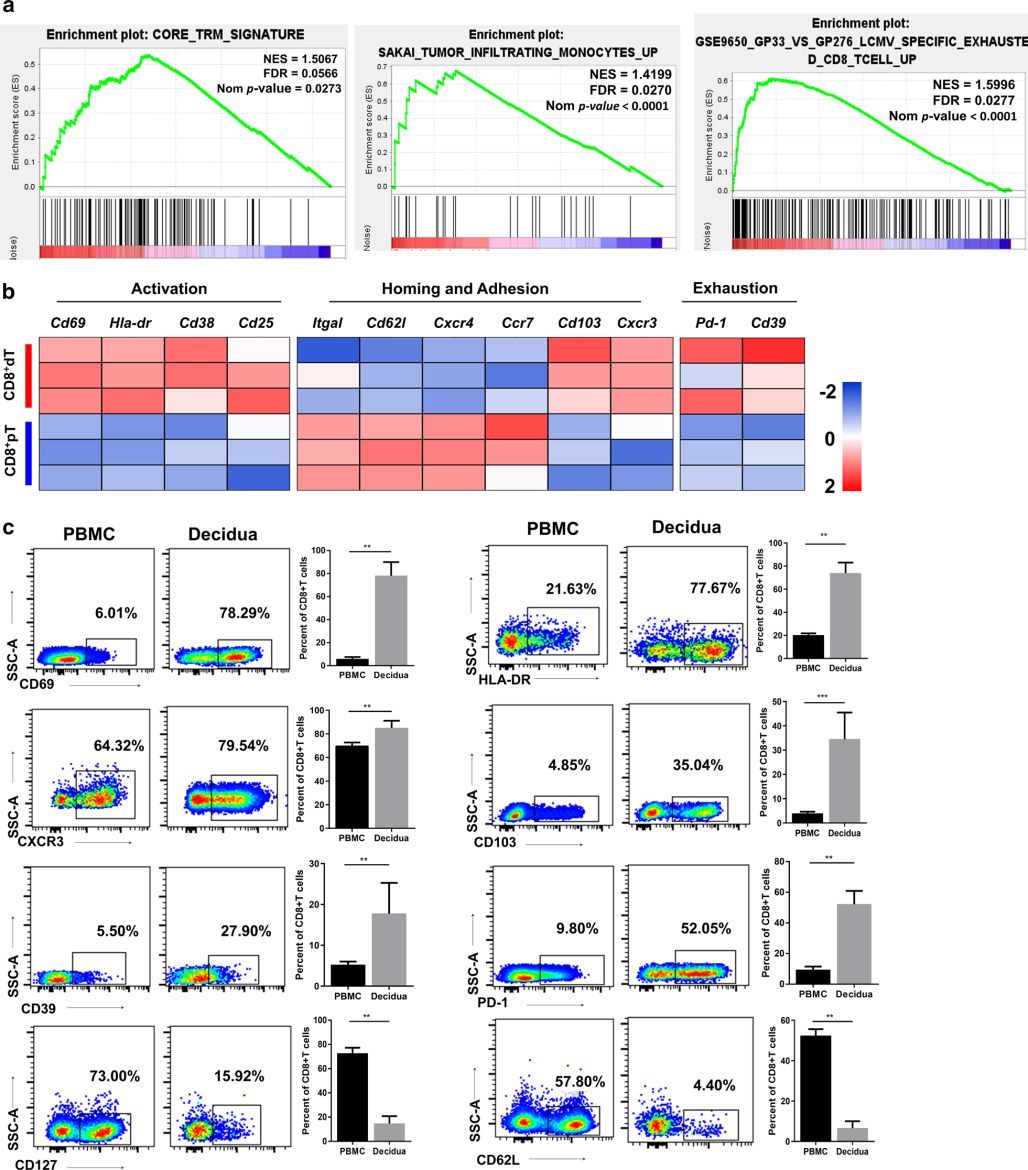
Combined residency and exhaustion signatures of CD8^+^dT cells. **a** GSEA analysis with gene sets of core T_RM_ signature, tumor-infiltrating monocytes and LCMV-specific exhausted CD8^+^T cells, respectively. NES, normalized enrichment score; FDR, false discovery rate; Nom, nominal. **b** Heat map of selected genes that are expressed differentially between CD8^+^dT cells and CD8^+^pT cells; gene expression was row normalized. **c** Representative FACS plots (Left) and percentages (Right) of the activation markers CD69, HLA-DR, tissue residency associated marker, CD103, CXCR3, and the coinhibitory molecule PD-1, as well as the CD39 molecule expressed on freshly isolated CD8^+^pT cells and CD8^+^dT cells. Graphs depict data of six to ten samples in each group; paired *t*^−^*test*, **p = < 0.01, ***p = < 0.005

Next, we explored the tissue residency signature of CD8^+^dT cells using core T_RM_ cell transcriptional signature genes that are computational integration of CD8^+^ T_RM_ gene-expression datasets from small intestine intraepithelial lymphocytes, the kidney, lung, skin, and brain [36]. The results of GSEA performed with a core T_RM_ cell signature gene set revealed a positive correlation, suggesting that T_RM_ cells share a residency signature with CD8^+^dT cells compared with CD8^+^pT cells (Fig. 3a). As T_RM_ cells and CD8^+^ TILs have a common core residency gene program, we performed GSEA analysis using genes that are upregulated in tumor-infiltrating monocytes which comprising tumor-associated macrophages, T lymphocytes, and antigen-presenting cells and NK cells [37], and we found a significant enrichment in upregulated genes of CD8^+^dT cells (Fig. 3a). Moreover, as recent research has described dysfunctional and exhausted signatures in tumor-infiltrating CD8^+^T cells [12], we analyzed the correlation of exhaustion signatures in CD8^+^dT cells with those in tumor-infiltrating CD8^+^T cells and found a positive correlation (Fig. 3a).

The analysis of a wide range of T cell activation markers (e.g., *Cd69*, *Hla*-*dr*, *Cd38*, and *Cd25*) and tissue homing and adhesion related genes (e.g., *Cxcr3* and *Cd103*) were upregulated in CD8^+^dT cells, whereas molecules responsible for blood and lymph node circulation (e.g., *Itgal*, *Cd62l*, *Cxcr4,* and *Ccr7*) increased in CD8^+^pT cells. CD8^+^dT cells upregulated the expression of exhaustion-related molecules (e.g., *Pd*-*1*, *Cd39*; Fig. 3b). We further analyzed levels of associated proteins using flow cytometry. The expression of selected activation markers (CD69, HLA-DR), tissue resident molecules (CXCR3, CD103), and T cell exhaustion–associated molecules (PD-1, CD39) was increased significantly in CD8^+^dT cells compared with CD8^+^pT cells (Fig. 3c). However, expression of the long-living memory T cell marker CD127 and the lymph nodes homing molecule CD62L was increased significantly in CD8^+^pT cells compared with CD8^+^dT cells (Fig. 3c).

### Decidual CD8^+^ T cells were selectively dysfunctional and not activated in response to autologous trophoblasts

**Fig. 4:**
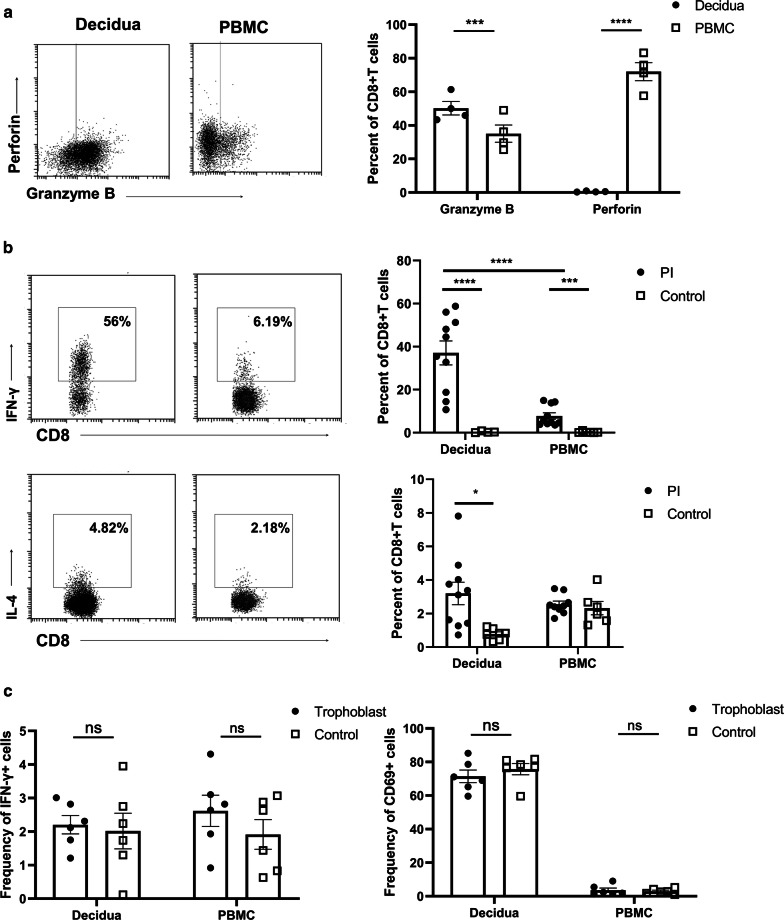
Detection of Intracellular effector molecules in CD8^+^dT cells and their activation after coculturing with autologous trophoblasts. **a** Representative FACS plots (Left) and percentages (Right) of the expression of intracellular granzyme B and perforin in total CD8^+^pT cells and CD8^+^dT cells; **b** IFN-γ, IL-4 expression in total CD8^+^pT cells and CD8^+^dT cells stimulated for 6 h with PMA/Ionomycin (1 μg/mL); **c** Lymphocytes from the decidua and PBMC were cultured separately with autologous trophoblasts for up to 24 h and were then tested for expression of CD69 and IFN-γ. Graphs show the data of four to ten samples in each group; a paired *t*-*test* was used to calculate the p-values, *p = < 0.05, ***p = < 0.005, ****p = < 0.0001

T cell exhaustion was not only associated with high expression of coinhibitory molecules, but also with reduced cytotoxic mediators and cytokines upon stimulation. In accordance with enriching exhaustion signatures, CD8^+^dT cells did not express the cytolytic molecule perforin but expressed significantly higher levels of granzyme B than did CD8^+^pT cells (Fig. 4a). Furthermore, the production of intracellular granzyme B in CD8^+^dT cells was upregulated upon activation/stimulation with PMA and ionomycin (Additional file 1: Figure S2a). Stimulation with PMA and ionomycin increased significantly the levels of intracellular IFN-γ in both CD8^+^dT cells and CD8^+^pT cells, and there was higher production of intracellular IFN^−^γ in CD8^+^dT cells following PMA and ionomycin stimulation than there was in in CD8^+^pT cells (Fig. 4b, Additioanl file 1: Figure S2a**)**. In addition, PMA and ionomycin treatment upregulated intracellular IL-4 secretion significantly in CD8^+^dT cells, but it had no effect on intracellular IL-4 secretion in CD8^+^pT cells (Fig. 4b). The antigen specificity of CD8^+^dT cells and their ability to recognize and respond to fetal antigens expressed by trophoblast cells is a key question that is, as yet, unresolved [38]. We determined the potential of CD8^+^T cells to activate in response to trophoblast cells through culturing CD8^+^dT cells and CD8^+^pT cells for 24 h in either the presence or absence of trophoblast cells from the same participant. Coculturing CD8^+^T cells with autologous trophoblast cells did not induce activation of either CD8^+^dT cells or CD8^+^pT cells, as revealed by invariable expression of the TCR activation marker CD69 and intracellular IFN-γ, whereas increased IFN-γ expression was detected in CD8^+^dT cells when they were cocultured with trophoblast from different individuals (data not shown).

### CD69^+^CD103^+^ T_RM_ CD8^+^dT cells were functionally impaired and DSCs can upregulate CD103 expression of CD8^+^pT cells

**Fig. 5:**
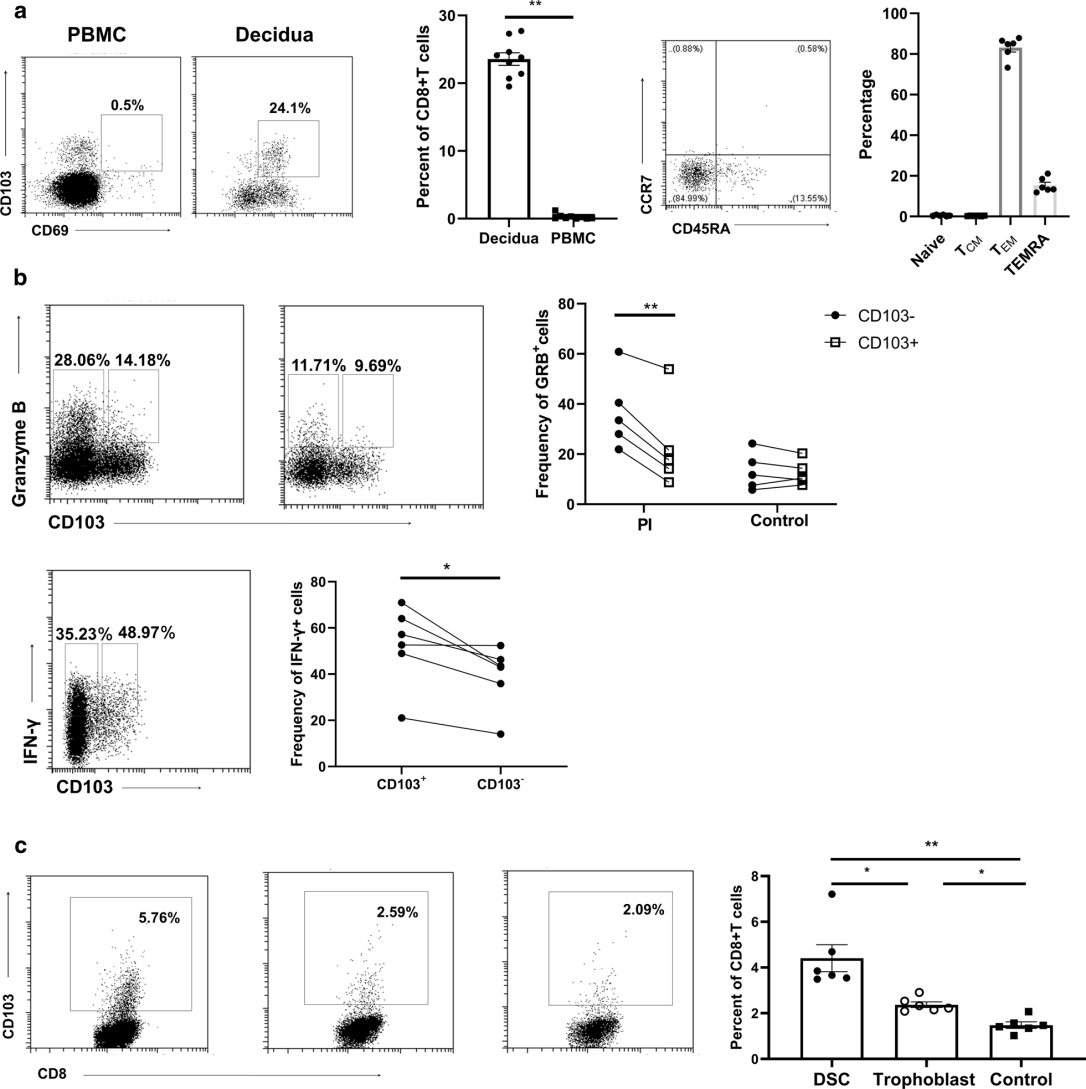
Upregulation of CD103 by decidual stromal cells and effector molecule expression in CD103^+^dT cells. **a** Representative FACS plots and percentages of expression of CD69 + CD103 + cells in CD8^+^pT cells and CD8^+^dT cells. Percentages of CD69 + CD103^+^ cells are depicted within four subpopulations of CD8^+^Tcell; **b** CD103 expression of CD8^+^pT cells cocultured with DSCs and trophoblasts, respectively, for three days and analyzed by flow cytometry; **c** Representative FACS plots (Left) and percentages (Right) of the expression of intracellular granzyme B and IFN-γ in CD103^+^ and CD103 − CD8^+^dT cells stimulated for 6 h with PMA/Ionomycin (1 μg/mL). Graphs show the data of six to ten samples in each group; a paired t-test was used to calculate the p-values, *p = < 0.05, ***p = < 0.005, ****p = < 0.0001. CD103 expression of CD8^+^pT cells cocultured with DSCs and trophoblasts, respectively, for three days and analyzed by flow cytometry. Graphs show the data of six samples in each group; a paired t-test was used to calculate the p-values, *p = < 0.05, **p = < 0.01

As gene expression analysis of CD8^+^dT cells demonstrated enrichment in core tissue resident signature genes and expression of CD69 and CD103 are believed to be attributed to residency of CD8^+^ T_RM_ cells in tissues despite a lack of persistent antigens, CD69 has been shown to have tissue-retention functions in the lymph nodes via the sequestration of the sphingosine-1-P receptor (S1PR) that mediates egress of T cells and is required for TRM retention in the skin, and CD103 adheres to E-cadherin, retaining the cells near the epithelial surface [39–42], we assessed expression of CD69 and CD103 in CD8^+^dT cells and CD8^+^pT cells. The percentage of CD69^+^CD103^+^ double positive cells in CD8^+^dT cells (19.5%–27.7%) increased significantly compared with that in CD8^+^pT cells, in accordance with the transcriptional signature of CD8^+^dT cells (Fig. 5a). However, we detected CD69^+^CD103^+^ cells rarely in CD8^+^pT cells (0.0%–1.21%), which was indicative of the circulating character of CD8^+^pT cells (Fig. 5a). As we anticipated, most CD69^+^CD103^+^ cells were effector memory cells (Fig. 5a). As CD8^+^dT cells also exhibited a combined transcriptional signature of tissue residency and exhaustion, we further evaluated their residency signature and effector responses upon stimulation. Treatment with PMA and ionomycin reduced the level of granzyme B significantly in CD103^+^CD8^+^dT cells compared with that in CD103^−^CD8^+^dT cells (Fig. 5b), suggesting compromised cytolytic ability of resident CD103^+^CD8^+^dT cells. Interestingly, there was a significantly higher level of intracellular IFN-γ in CD103^+^CD8^+^dT cells than in CD103^−^CD8^+^dT cells (Fig. 5b), whereas there was no difference in IL-4 production between the two cell subsets (Additional file 1: Figure S3a). In addition, to examine interactions of the fetal–maternal interface microenvironment with CD8^+^pT cells, we cocultured CD8^+^pT cells with DSCs and trophoblasts. This significantly increased CD103 expression in CD8^+^pT cells, whereas coculturing with DSCs induce the upregulation of CD103 in CD8^+^pT cells more significant than trophoblast cells (Fig. 5c).

## Discussion

Decidual T cells are composed of very heterogenic subset of T cells containing accumulated γδ T cells as well as activated and regulatory T cells, which are highly specialized and have major differences as compared with peripheral blood T cells. Although CD8^+^ dT cells are thought to be delicately tempered during healthy pregnancy, limited data are available on the phenotypic and functional characteristics of these cells, which remain controversial [43, 46]. In this study, we discovered impaired effector functions of CD8^+^dT cells by dissecting their differentiation status and functional attributes, detailed phenotypes and transcriptional assessments further revealed their dysfunctions and tissue residency signatures. Furthermore, we showed that both CD8^+^dT and CD8^+^pT cells cannot be activated by autologous trophoblast cells, whereas DSCs can increase the CD8^+^pT residency signature. Our findings add to the knowledge of the biology of CD8^+^dT cells and how they balance protective immunity with maternal–fetal tolerance, which can be used as a target for predicting adverse pregnancy outcomes.

CD8^+^T cells exhibit high heterogeneity with various cell subsets. Our study revealed that decidua contain a significant reduced percentage of naïve cells and T_CM_, whereas a significant increased percentage of T_EM_. Terminally differentiated T_EMRA_ were equivalent between CD8^+^dT and CD8^+^pT. T_EM_ and T_EMRA_ of CD8^+^dT cells show increased CD27^+^CD28^−^ subsets compared with CD8^+^pT, CD27 and CD28 that are costimulatory receptors involved in the generation of Ag-primed cells and regulation of T cell activation, respectively [34, 35], CD27^+^CD28^−^ subsets are intermediate with partial effector functions and replicative history [33]. The partial dysfunction of CD8^+^dT cells was validated by their absence of perforin expression, and higher granzyme B, IFN-γ, and IL-4 production. Progesterone can promote maternal–fetal tolerance by inducing a specific cytokine profile of maternal T cells [44]. Particularly, progesterone is known to induce increased levels of IL-4 in maternal CD4^+^ and CD8^+^ T cells and limits their cytotoxicity [45]. Thus, CD8^+^dT have an unusual cytokine profile that may potentially relate to local effects of progesterone [44, 46]. However, we found a high level of perforin mRNA in CD8^+^dT RNA-seq data (data not shown), indicating that posttranscriptional regulation was responsible for absence of perforin expression. These findings indicated that CD8^+^dT cells may undergo a specific type of cell differentiation that contributes to meeting the competing requirements for fetal tolerance and protection from infections.

The presence of highly differentiated CD8^+^dT cells implies that they may be induced by fetal alloantigen from trophoblasts. Because trophoblasts lack HLA-A, -B, -DR, -DQ, and -DP molecules, HLA-C and minor histocompatibility antigen are the only targets to which CD8^+^ T cells respond [47, 48]. A study in mice revealed that maternal naïve T cells primed by fetal antigens differentiated into long-lived PD-1^+^CD8^+^T cells with selective silencing of effector function [49]. The study provides clues that pregnancy elicits CD8^+^T cells with similarity to the dysfunctional and exhausted T cells found in chronic infection and TILs, which also feature selective silencing of effector function and upregulation of coinhibitory molecules. Our study on CD8^+^dT cell gene-expression profiles demonstrated significant enrichment in T cell exhaustion, which was confirmed by high protein expression of the exhaustion-related molecules PD-1 and CD39. The combined results of transcription, protein expression, and function analysis provided further evidence of CD8^+^dT dysfunction. In our previous data, the blockade of PD-1 and Tim-3 resulted in decreased in vitro proliferation and IL-4 cytokine production, whereas it increased trophoblast killing and IFN-γ producing capacities of CD8^+^dT cells [50]. In addition, trophoblasts can indirectly influence the immune system during pregnancy via their expression of soluble and cell-surface-associated immunomodulatory molecules. We did not detect the activation of CD8^+^dT or CD8^+^pT cells when they were cocultured with trophoblasts. As CD8^+^pT cells are mainly naïve T cells and CD8^+^dT are antigen-primed effector or effector memory T cells, the unresponsiveness of CD8^+^pT when cocultured with trophoblasts might be due to their lack of fetal antigen specificity. Studies have indicated that the dendritic cells resident in the decidua are constrained in their ability to leave the tissue and migrate to adjacent lymph nodes where they can activate circulating T cells [43]. Whether the failure of CD8^+^pT cells to respond to autologous trophoblasts is due to the lack of antigen specificity to fetal antigens or to immunomodulatory molecules of trophoblasts needs further research.

T_RM_ cells are a distinctive effector memory population with a unique phenotype influenced by their localization within tissue [51–53]. Although CD8^+^dT cells have been shown to express CD103, it is still unclear if they are true tissue resident T cells. Our study showed that gene expression of CD8^+^dT cells was also enriched in the core residency gene program, though it is still unclear whether they are fetal antigen-specific. T_RM_ cells constitute as a first line of defense for optimal immunity against pathogens [54, 55]. Upon recognition of invading pathogens, T_RM_ cells rapidly release IFN-γ and other proinflammatory cytokines and chemokines. However, in vivo, they are poorly cytolytic, and they fail to expand after encountering antigens [56, 57]. As our data shows, T_RM_ cells infiltrating the decidua share similar functions, with less granzyme B but higher expression of IFN-γ following stimulation, this may indicate their capability of pathogen defending. The silencing of chemokines in decidua can inhibit the migration of maternal effector T cells to the maternal–fetal interface [58]. Our findings also demonstrate that DSCs and trophoblasts can affect the residency signatures of CD8^+^pT cells by upregulating the expression of CD103. Exposure to TGF-β has been shown to lead to CD103 expression in T_RM_ cells [59, 60], which often reside near epithelial cells that express E-cadherin. The components at the maternal–fetal interface including embryonic trophoblasts, maternal DSCs, and decidual lymphocytes can produce substantial TGF-β [59, 61–63], which may explain why CD103-expressing T_RM_ cells are resident in the uterus during pregnancy. A recent study has demonstrated that the recall responses of brain T_RM_ cells can induce reactive gliosis; however, the function of resident CD8^+^dT cells remains to be elucidated under the scenario of antigen–specific reactivation. The question of whether adverse stimulation such as infections or aberrant cytokines can destabilize the tolerance microenvironment via upregulating CD8^+^dT cell cytotoxicity and contribute to fetal rejection is central to provide new clues for immunotherapy of pregnancy complications, such as miscarriages and preterm birth.

Our findings reinforce the concept that CD8^+^ T cells were delicately tempered during early pregnancy, and the local modulation of decidual infiltrating CD8^+^ T cells may contribute to the maintenance of maternal–fetal tolerance. Improved understanding of the regulation of the immunological balance at the maternal–fetal interface by cellular processes distinct to maternal immune cells, metal trophoblasts, and maternal hormonal changes is likely to have substantial implications for various areas of clinical immunology.

## Supplementary information

**Additional file 1: Figure S1.** TCM and Tnaive were analyzed for CD27 versus CD28 staining. **a** The percentage of four subsets divided by CD27 and CD28. **Figure S2.** CD8 + dT were treated with PMA/Ionomycin stimulation. **a** Percentage of intracellular granzyme B expression. **b** The gating of IFN-γin CD8 + Tcellson multiple patients. **Figure S3**. A Percentage of intracellular IL-4 expressionin CD103 + versus CD103-CD8 + T cells.

## Data Availability

All data generated or analyzed during this study are included in this published article and its supplementary information files.
