# Supplementary material for: Decidual CD8+T cells exhibit both residency and tolerance signatures modulated by decidual stromal cells
Source: J Transl Med. 2020 Jun 1;18:221. doi: 10.1186/s12967-020-02371-3 (PMC7268777; doi:10.1186/s12967-020-02371-3)
Supplement: Supplementary file 1 — Additional file 1: Figure S1. TCM and Tnaive were analyzed for CD27 versus CD28 staining. a The percentage of four subsets divided by CD27 and CD28. Figure S2. CD8 + dT were treated with PMA/Ionomycin stimulation. a Percentage of intracellular granzyme B expression. b The gating of IFN-γin CD8 + Tcellson multiple patients. Figure S3. A Percentage of intracellular IL-4 expressionin CD103 + versus CD103-CD8 + T cells. [file 12967_2020_2371_MOESM1_ESM.doc]

**Additional file 1**

**
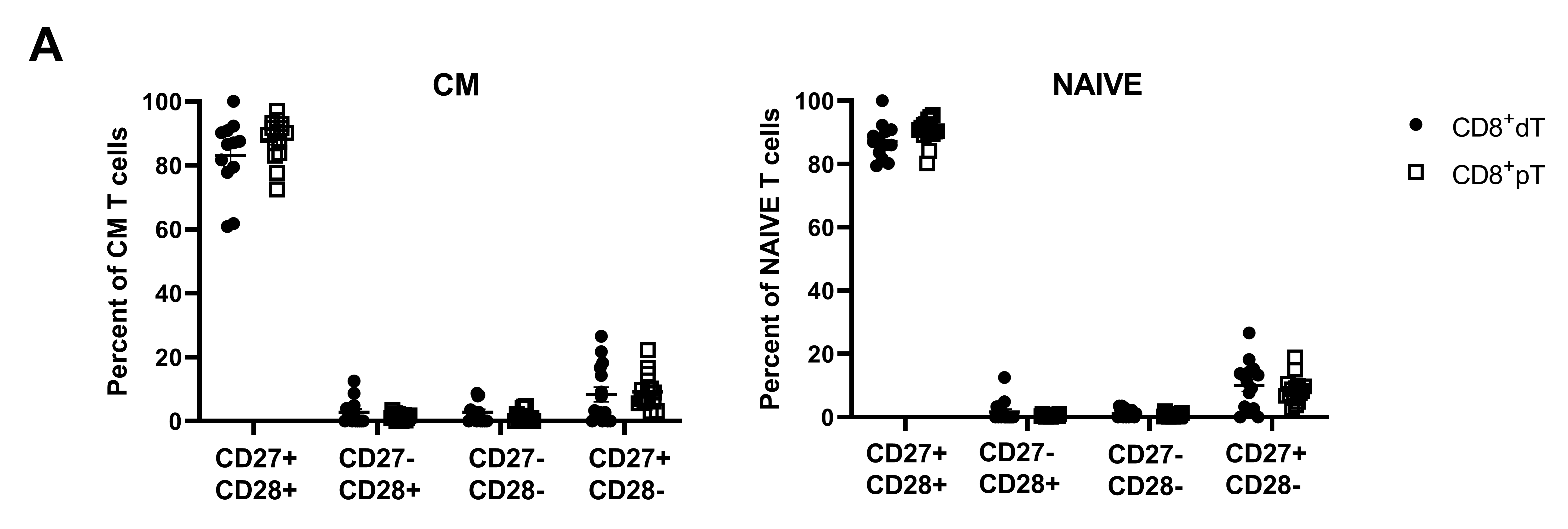
**

**Figure S1.** T_CM_ and T_naive_ were analyzed for CD27 versus CD28 staining. **(A)** The percentage of four subsets divided by CD27 and CD28.

**Figure S2.**
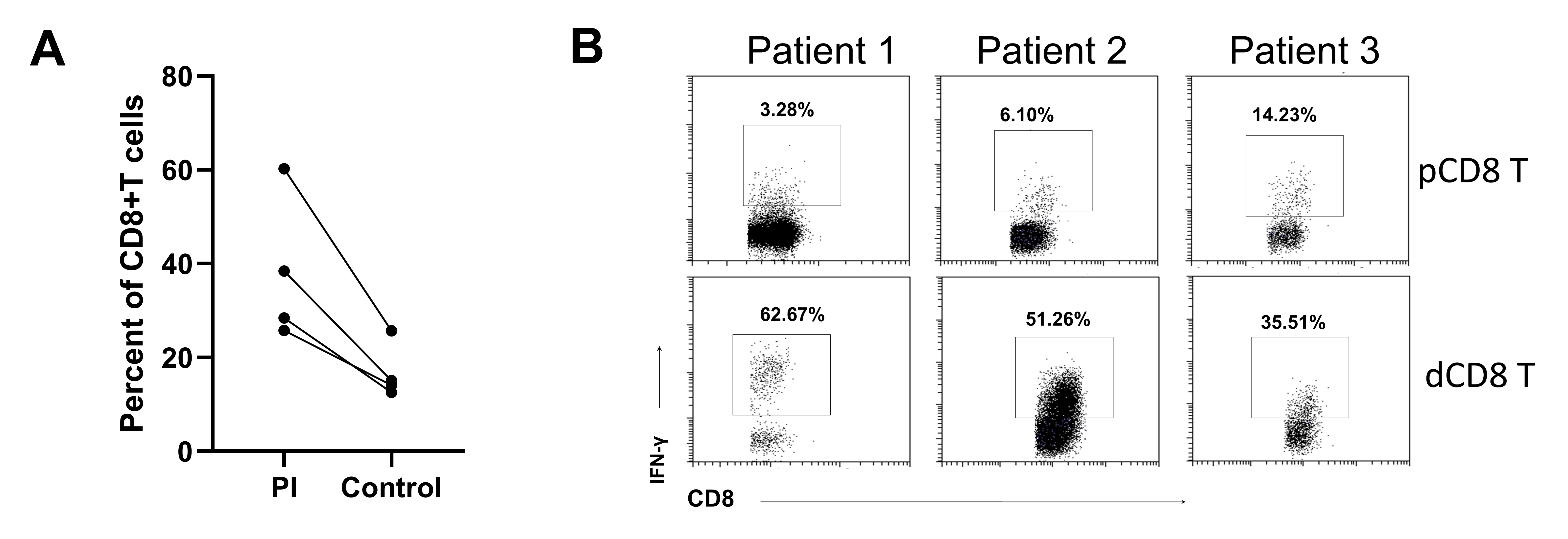
CD8^+^ dT were treated with PMA/Ionomycin stimulation. **(A)** Percentage of intracellular granzyme B expression. **(B)** The gating of IFN-γ in CD8^+^ T cells on multiple patients.

**Figure S3. (A)** Percentage of intracellular IL-4 expression in CD103^+^ versus CD103^-^CD8^+^
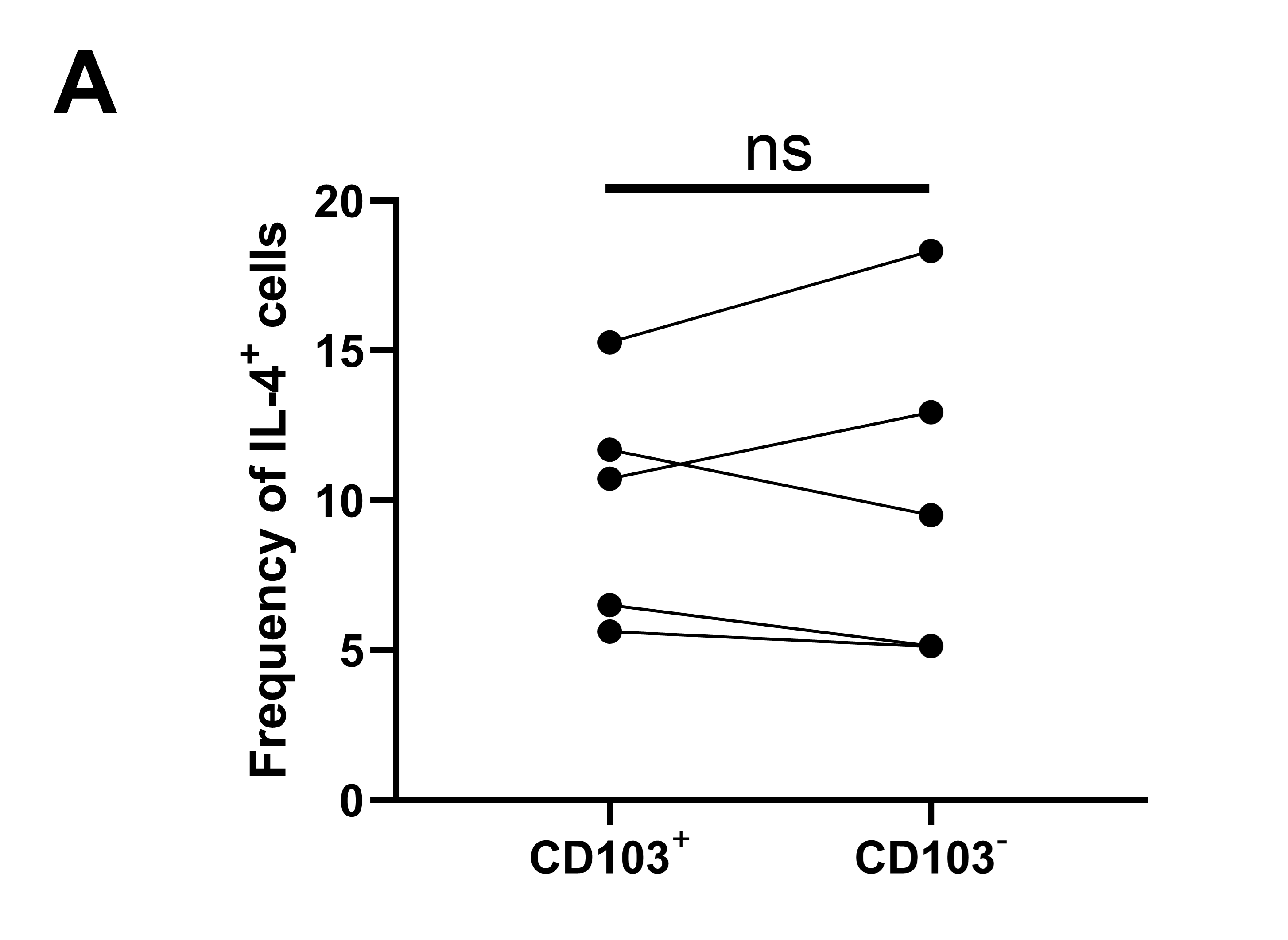
 T cells.
